# Effect of a progesterone-releasing intravaginal device (PRID) for 8 days during a modified Ovsynch protocol on pregnancy outcomes in lactating Holstein cows

**DOI:** 10.3168/jdsc.2022-0314

**Published:** 2023-02-24

**Authors:** M. Hölper, L. Bretzinger, F. Randi, W. Heuwieser, S. Borchardt

**Affiliations:** 1 Clinic for Animal Reproduction, Faculty of Veterinary Medicine, Freie Universitaet Berlin, Koenigsweg 65, 14163 Berlin, Germany; 2 Ceva Santé Animale, 33500 Libourne, France

## Abstract

Our objective was to evaluate the effect of a progesterone-releasing intravaginal device (PRID) in a 7-d Ovsynch protocol on pregnancy per artificial insemination (P/AI) and pregnancy loss, compared with a standard 7-d Ovsynch protocol without progesterone supplementation. We hypothesized that progesterone supplementation during an Ovsynch protocol would increase P/AI and decrease pregnancy loss. Data were collected on lactating Holstein cows (n = 716) that either received a 7-d Ovsynch protocol (control: d 0, 100 µg of GnRH; d 7, 500 µg of cloprostenol; d 9, µg of GnRH; n = 360) or a modified Ovsynch protocol with addition of a PRID (PRIDsynch; d 0, 100 µg of GnRH + PRID; d 7, 25 mg of dinoprost; d 8, PRID removal; d 9, 100 µg of GnRH; n = 356). All cows received timed artificial insemination (TAI) approximately 16 h after the second GnRH treatment. Pregnancy diagnosis was performed via ultrasonography on d 38 ± 3 after TAI and rechecked on d 80 ± 7 after TAI. Reproductive performance differed between treatments, with PRIDsynch cows having greater (38.9%) P/AI compared with control cows (31.7%) at d 38 ± 3 and also at d 80 ± 7 (34.6% vs. 28.9%, for PRIDsynch and control cows, respectively). Pregnancy loss did not differ among treatments.

## Highlights

• Insertion of a PRID from day 0 until day 8 of the protocol increased pregnancy per artificial insemination in dairy cows submitted to a 7-day Ovsynch protocol.
• Pregnancy loss was not affected by progesterone supplementation.
• We observed no interaction between treatment and season, parity, or number of artificial inseminations.

## Graphical Abstract

**Figure:**
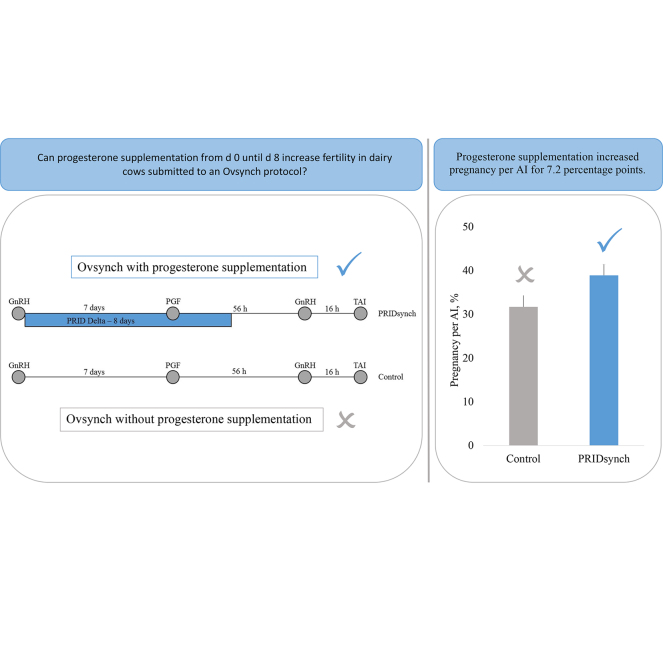
**Summary:** The objective of this study was to compare the effect of a progesterone-releasing intravaginal device (PRID) in a 7-day Ovsynch protocol on pregnancy per artificial insemination and pregnancy loss to a standard 7-day Ovsynch protocol without progesterone supplementation. Progesterone supplementation increased pregnancy per artificial insemination by 7.2 percentage points.

Improving fertility outcomes in protocols for timed artificial insemination (**TAI**) has been a central part of reproductive research in recent years (Stevenson and Britt, 2017). The initial Ovsynch protocol (Pursley et al., 1997), allowing for AI without the necessity of heat detection, consisted of a GnRH treatment, followed by PGF_2α_ at d 7 of the protocol and a final GnRH treatment at d 9, approximately 16 h before TAI. Different presynchronization regimens [e.g., Presynch-Ovsynch (Moreira et al., 2001), Double-Ovsynch (Souza et al., 2008), or G6G (Bello et al., 2006)] and modifications of timing (Brusveen et al., 2009) and dose (Giordano et al., 2013) of PGF_2α_ treatments were developed to optimize the hormonal environment at initiation and during an Ovsynch protocol. Another approach to improve fertility in TAI protocols is the supplementation of progesterone (**P4**; El-Zarkouny and Stevenson, 2004) by insertion of a progesterone-releasing intravaginal device (**PRID**). Lactating dairy cows without a mature corpus luteum (**CL**) at the start of an Ovsynch protocol (i.e., at the first GnRH treatment, **G1**) experience insufficient concentrations of P4 during follicular development, which causes reduced reproductive performance (Stevenson et al., 2006; Wiltbank et al., 2014). Approximately 30% of first AI cows submitted to TAI protocols (Stevenson et al., 2008) and 22 to 46% of cows resynchronized for AI (Fricke et al., 2003) do not bear a functional CL at protocol initiation, leading to low P4 concentrations at G1. This compromises oocyte quality (Santos et al., 2016) due to suboptimal follicular development (Cerri et al., 2011), which results in reduced P/AI and greater pregnancy losses (Wiltbank et al., 2011; Bisinotto et al., 2015b). Additionally, cows suffering from low preovulatory P4 concentrations are more susceptible to double ovulations and greater twinning rates (Carvalho et al., 2019). Insufficient P4 levels may also affect fertility in high-producing dairy cows with a CL at protocol initiation, due to an increased hepatic P4 metabolism (Wiltbank et al., 2006). Several studies have addressed the positive effects of progesterone during the first 7 d of an Ovsynch protocol (Bisinotto et al., 2015b); however, the effect of an extended P4 supplementation for 8 d during an Ovsynch protocol on reproductive performance is only occasionally described (Xu and Burton, 2000).

Therefore, the objective of this study was to compare the standard 7-d Ovsynch protocol with a modified protocol including a PRID device. We hypothesized that P4 supplementation by insertion of a PRID device for 8 d would increase P/AI and decrease pregnancy loss in lactating Holstein cows submitted to an Ovsynch protocol.

All experimental procedures were approved by the Institutional Animal Care and Use Committee of the Freie Universität Berlin (reference number 2347-A-3–2-2020).

The experiment was conducted from October 2020 until July 2021. Lactating Holstein cows (n = 716; 236 primiparous and 480 multiparous cows) from one commercial dairy farm in Northeast Germany were used in this study. The farm comprised 1,600 Holstein Friesian cows with an average 305-d ECM yield of 9,809 kg. Lactating cows were housed in a freestall barn with slatted floors and beds equipped with rubber mats. Group composition was dynamic, with cows entering and leaving the study barn depending on their calving dates. Cows were fed a TMR twice a day in a 12-h interval with free access to feed and water. The rations were formulated to meet or exceed the requirements according to the NRC (2001). Lactating cows were milked 2 times daily, starting at 0730 and 2000 h.

The farm's reproductive management consisted of a Presynch-Ovsynch protocol for first AI, with the possibility of AI either at detected estrus after the first PGF_2α_ (50 ± 3 DIM) or the second PGF_2α_ treatment (64 ± 3 DIM). Cows not detected in estrus during this presynchronization received TAI at the end of the following Ovsynch protocol (86 ± 3 DIM; Figure 1). Cows were assigned to treatment for first AI after presynchronization. After nonpregnancy diagnosis, open cows were also assigned to treatment. Lactating Holstein cows received an Ovsynch protocol with addition of a PRID from d 0 until removal at d 8 as part of a larger study (Hölper et al., 2023) conducted by our research team. All cows not enrolled in that previous study were treated with a standard 7-d Ovsynch protocol by the AI technician as part of the farm's reproductive procedure. The scope of the present study was a comparison of an 8-d P4-based Ovsynch protocol (PRIDsynch: d 0, 100 µg of GnRH + PRID; d 7, 25 mg of dinoprost; d 8, PRID removal; d 9, 100 µg of GnRH) with a standard 7-d Ovsynch protocol (control: d 0, 100 µg of GnRH; d 7, 500 µg of cloprostenol; d 9, 100 µg of GnRH). Cows that lost the PRID before scheduled removal (n = 20), cows that left the herd before pregnancy diagnosis (n = 16), and cows that were not treated or inseminated according to the protocol (n = 14) were excluded. For the PRID group (n = 356), GnRH (gonadorelin diacetate tetrahydrate; Ovarelin 50 µg/mL), PGF_2α_ (dinoprost trometamol; Enzaprost T 5 mg/mL), and progesterone devices (progesterone; PRID Delta 1.55 g) were from Ceva Santé Animale. For the control group (n = 360), GnRH (gonadorelin acetate; Gonavet Veyx Forte 50 µg/mL) and PGF_2α_ analog (cloprostenol sodium; PGF Veyx Forte 250 µg/mL) were from Veyx Pharma GmbH. Approximately 16 h after the second GnRH treatment, all cows received TAI by a single trained AI technician.

**Figure 1:**
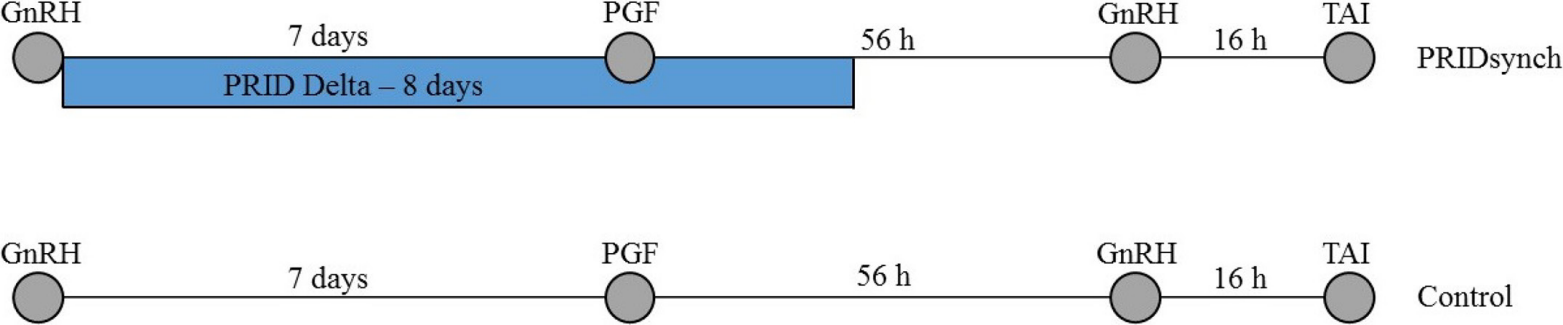
Schematic representation of the study design. Control cows (n = 360) received a standard 7-d Ovsynch protocol (d 0, 100 µg of GnRH; d 7, 500 µg of cloprostenol; d 9, 100 µg of GnRH). PRIDsynch cows (n = 356) received an Ovsynch protocol with an addition of a progesterone-releasing intravaginal device (PRID) from d 0 until removal at d 8 (d 0, 100 µg of GnRH + PRID; d 7, 25 mg of dinoprost; d 8, PRID removal; d 9, 100 µg of GnRH). All cows received TAI approximately 16 h after the second GnRH treatment. PGF = prostaglandin F_2α_; TAI = timed artificial insemination.

Pregnancy diagnosis was performed by transrectal ultrasound examination d 38 ± 3 after AI and reconfirmed d 80 ± 7 after AI by a veterinarian using a portable ultrasound device (Easi-Scan:GO, IMV Imaging) equipped with a 7.5-MHz linear-array transducer. A positive pregnancy diagnosis was based on visualization of an embryo with a heartbeat. Nonpregnancy diagnosis was based on absence of pregnancy at the day of examination or a rebreeding to an estrus before pregnancy diagnosis. Pregnancy loss included cows that experienced pregnancy loss from d 38 to 80.

The results of pregnancy diagnosis and additional breeding information were obtained from the herd management software (HerdeW, dsp-Agrosoft GmbH) and transferred to Excel (Office 2016, Microsoft Deutschland Ltd.). All data were analyzed using SPSS for Windows (version 25.0, IBM Corp.). Logistic regression models for P/AI and pregnancy loss analysis were built using the GENLINMIXED procedure of SPSS. Cow was the experimental unit. Model building was conducted as recommended by Dohoo et al. (2009), where each parameter was first analyzed separately in an univariable model. Only parameters resulting in univariable models with *P* ≤ 0.10 were included in the final mixed model. Selection of the model that best fit the data was performed by using a backward stepwise elimination procedure that removed all variables with *P* > 0.10 from the model. The initial model included the following explanatory variables as fixed effects: treatment (control vs. PRIDsynch), parity (primiparous vs. multiparous), season of AI (winter from December 1 to February 28, spring from March 1 to May 31, summer from June 1 to July 31, and autumn from October 1 to November 30), and AI number (first TAI vs. subsequent TAI), as well as interactions between these variables. Treatment and parity were forced to stay in the final model, regardless of their significance level. The final model consisted of treatment, parity, and season with no interaction between these variables.

A Bonferroni adjustment was used to account for multiple comparisons. Variables were declared to be significant when *P* < 0.05. A statistical tendency was declared when *P* ≥ 0.05 and *P* ≤ 0.10.

Descriptive data are summarized in Table 1. Parity distribution differed (*P* = 0.003) among treatments [control (101/360) vs. PRIDsynch (135/356)]. Average milk production did not differ among treatments (*P* = 0.390; PRIDsynch: 34.9 ± 7.7 kg; control: 35.5 ± 7.0 kg). The proportion of cows receiving first postpartum AI did not differ (*P* = 0.347) among treatments. The proportion of cows receiving second AI and greater did not differ (*P* = 0.289) among treatments. Days in milk at enrolment did not differ among treatments (*P* = 0.411).

**Table 1:** Comparison of descriptive data for cows enrolled in this experiment and comparison of pregnancy per artificial insemination [P/AI, % (number of pregnant cows/number of all cows)] and pregnancy loss between treatments

Item	Treatment^1^		*P-*value
	Control	PRIDsynch	
DIM at enrolment (± SD)	145.9 ± 82.7	141.6 ± 80	0.411
Primiparous cows, % (n/N)	28.1 (101/360)	37.9 (135/356)	0.003
First service, % (n/N)	45.3 (197/360)	43.5 (201/356)	0.347
P/AI, % at d 38 ± 3 (n/N)	31.7 (115/360)	38.9 (148/356)	0.014
P/AI, % at d 80 ± 7 (n/N)	28.9 (103/358)	36.3 (137/354)	0.004
Pregnancy loss, % (n/N)	8.8 (10/113)	6.2 (9/146)	0.279

1 Lactating Holstein cows were randomly assigned to receive an Ovsynch protocol with addition of a progesterone-releasing intravaginal device (PRID) from d 0 until removal at d 8 (PRIDsynch; d 0, 100 μg of GnRH + PRID; d 7, 25 mg of dinoprost; d 8, PRID removal; d 9, 100 μg of GnRH) or a standard 7-d Ovsynch protocol (control: d 0, 100 μg of GnRH; d 7, 500 μg of cloprostenol; d 9, 100 μg of GnRH). All cows received timed artificial insemination (TAI) approximately 16 h after the second GnRH treatment. Percentages for P/AI were derived from the GENLINMIXED model including treatment, parity, and season, with no interaction between these variables.

Pregnancy per AI at d 38 ± 3 differed among treatments (*P* = 0.014). Cows receiving PRIDsynch had greater P/AI (38.9%) at d 38 ± 3 after AI (Table 1), compared with the control (31.7%). Pregnancy per AI at d 38 ± 3 after AI did not differ between primiparous and multiparous cows (*P* **=** 0.171). There was no treatment by parity interaction (*P* = 0.989).

Pregnancy per AI differed (*P* = 0.004) among treatments at d 80 ± 7 after AI, with PRIDsynch cows having greater (36.3%) P/AI compared with cows in the control group (28.9%). Pregnancy per AI differed (*P* = 0.044) among parity at d 80 ± 7 after AI, with primiparous cows having greater (34.6%) P/AI compared with multiparous cows (30.4%). There was, however, no interaction between treatment and parity (*P* = 0.670). Season had a significant influence on P/AI at d 38 ± 3 (*P* = 0.001) and d 80 ± 7 (*P* = 0.001). Lowest P/AI at d 38 ± 3 and d 80 ± 7 post AI was observed in summer (19.9% and 18.6%, respectively). Cows had the greatest P/AI at d 38 ± 3 and d 80 ± 7 post AI in spring and winter (43% and 39.7%, respectively). In August and September, no cows were enrolled in this study. Pregnancy loss did not differ (*P* = 0.279) among treatments (8.8% vs. 6.2% for control and PRIDsynch cows, respectively).

Results from this study support the use of P4 devices in Ovsynch protocols from d 0 until removal at d 8 in lactating Holstein cows. Our results show that inserting a PRID at the beginning of an Ovsynch protocol increased P/AI regardless of parity or number of AI.

Insufficient P4 concentrations during the follicular development are a major concern in lactating dairy cows submitted to TAI protocols (Bisinotto et al., 2014; Wiltbank et al., 2014). Lower P4 concentrations lead to an increased pulse frequency of LH (Endo et al., 2012) and premature meiotic resumption, ultimately decreasing oocyte quality (Revah and Butler, 1996). Moreover, decreased preovulatory blood P4 concentrations can alter endometrial morphology and secretory functions during early gestation (Shaham-Albalancy et al., 1997) and cause an increased release of PGF_2α_ in response to oxytocin in the subsequent cycle after AI (Cerri et al., 2011). Interestingly, previous research showed that interferon tau (IFN-τ) secretion on d 17 of gestation, associated with pregnancy establishment and maintenance, was not affected by follicular wave dynamics and therefore P4 concentrations during follicle growth (Carvalho et al., 2019; Bisinotto et al., 2022). These and our findings indicate that further research on the effects of progesterone regarding embryonic development and pregnancy establishment needs to be conducted.

Nonetheless, P4 concentrations of at least 2.0 to 3.0 ng/mL were described as beneficial regarding P/AI and pregnancy loss, restoring fertility of cows with no CL at the beginning of the protocol to the level of those cows initiated at diestrus (Bisinotto et al., 2013). In the above-mentioned studies, however, 2 controlled internal drug release (**CIDR**) devices with a P4 content of 1.38 g each were used to elevate progesterone concentrations. Using a single PRID device with a P4 content of 1.55 g from d 0 until d 8 of the protocol resulted in mean P4 concentrations of 2.68 and 4.34 ng/mL at d 7 of the protocol for cows without and with a CL at G1, respectively (Hölper et al., 2023). These findings are supported by previous studies on P4 supplementation with PRID devices, describing overall greater P4 concentrations and P/AI (van Werven et al., 2013) and a greater initial increase in P4 (Silva et al., 2021), compared with other intravaginal devices for P4 supplementation. Further research is warranted to investigate the influence of the surface area or material of the outer layer on P4 absorption.

In a meta-analysis by Bisinotto et al. (2015b), it was described that the insertion of a single P4 device can benefit reproductive performance, but mainly in cows without a CL on d 0 of the TAI protocol. The meta-analysis described an overall increase in P/AI of 3.4 percentage points on d 32 after AI, but a tendency for a decrease in pregnancy loss. In our study, an overall P/AI increase of 7.2 percentage points was achieved, whereas pregnancy loss was not affected by treatment. A direct comparison with our study seems difficult as only 3 out of 25 studies included in this analysis used a PRID device for progesterone supplementation and most of the studies in the meta-analysis removed the CIDR or PRID after 7 d compared with 8 d in our study.

Estrus expression on the day of TAI is generally beneficial for fertility (Santos et al., 2010). The extended time of P4 supplementation may contribute to greater fertility by reducing the proportion of cows with premature ovulation (Colazo et al., 2013) and estrus expression before TAI. This occurs in 5 to 7% of cows submitted to TAI protocols (Bisinotto et al., 2015a). An improved precision in the onset of estrus was observed by Xu and Burton (2000) when using a P4 device for 8 d. Research by Herlihy et al. (2013) confirms this statement and additionally shows a beneficial effect of P4 supplementation for 8 d during TAI protocols on P/AI in high-producing cows, independent of luteal status at protocol initiation, thus supporting our initial hypothesis. Furthermore, P4 supplementation increases the proportion of cows with a functional CL at d 11 to 14 after TAI (Chebel et al., 2010) indicating improved synchrony of ovulation. Stevenson et al. (2008) reported a decrease in ovulatory response to the first GnRH treatment when P4 was supplemented at the same time, thus impeding fertility by extending the period of follicle dominance. However, various studies did not show an association between the addition of P4 devices and a lack of ovulatory response after G1 (Bisinotto et al., 2010; Dewey et al., 2010). Although Stevenson (2016) described an association between greater P4 concentrations at G1 and a decreased ovulatory response after the first GnRH treatment, he also observed greater P/AI in cows with P4 concentrations ≥3 ng/mL at protocol initiation. This author concludes that greater P4 concentrations during follicular development may have a greater impact on fertility compared with the ovulatory response after G1.

One limitation of the study is the unequal percentage of primiparous cows in the 2 groups. Bamber et al. (2009) described a greater proportion of anovular cows within the first lactation. On the other hand, primiparous cows have better fertility as shown in multiple studies (Tenhagen et al., 2004; Fricke et al., 2014). Therefore, parity was forced to remain in the model. Considering this, the results of this study may be somewhat influenced by the greater proportion of primiparous cows in the PRIDsynch group. In addition, different types of prostaglandin were used in between the 2 groups. Although several studies did not show an effect of different products on fertility (Stevenson and Phatak, 2010; Baryczka et al., 2018), other studies described that the subtle differences between those variations may lead to a greater reproductive performance when using cloprostenol sodium (Pursley et al., 2012), which should be considered when interpreting the results of the present study.

Taking that into account, supplementation of progesterone by means of a PRID device from d 0 until d 8 in a 7-d Ovsynch protocol seemingly improved P/AI without effect on pregnancy loss. Further research needs to be conducted regarding the effect of prolonged P4 supplementation in Ovsynch protocols on reproductive performance and P4 concentrations. Furthermore, the effect of a CL at protocol initiation and estrus expression before TAI throughout the protocol needs to be addressed in future studies.
